# Kiss Y flap: a novel low-tension reconstruction technique after excision of pilonidal sinus

**DOI:** 10.1093/gastro/goag025

**Published:** 2026-03-22

**Authors:** Jianlei Liu, Xiaochun Zhang, Xiaorui Ye, Jun Wang, Yang Yang, Jiabo Gu, Chunxia Zhang, Xinyi Zhang, Heiying Jin

**Affiliations:** Department of Colorectal Surgery, The Second Affiliated Hospital of Nanjing University of Chinese Medicine, Nanjing, Jiangsu, P. R. China; Department of Colorectal Surgery, The Second Affiliated Hospital of Nanjing University of Chinese Medicine, Nanjing, Jiangsu, P. R. China; Second Clinical Medical College, Nanjing University of Chinese Medicine, Nanjing, Jiangsu, P. R. China; Department of Colorectal Surgery, The Second Affiliated Hospital of Nanjing University of Chinese Medicine, Nanjing, Jiangsu, P. R. China; Department of Colorectal Surgery, The Second Affiliated Hospital of Nanjing University of Chinese Medicine, Nanjing, Jiangsu, P. R. China; Department of Colorectal Surgery, The Second Affiliated Hospital of Nanjing University of Chinese Medicine, Nanjing, Jiangsu, P. R. China; Department of Colorectal Surgery, The Second Affiliated Hospital of Nanjing University of Chinese Medicine, Nanjing, Jiangsu, P. R. China; Department of Colorectal Surgery, The Second Affiliated Hospital of Nanjing University of Chinese Medicine, Nanjing, Jiangsu, P. R. China; Department of Colorectal Surgery, The Second Affiliated Hospital of Nanjing University of Chinese Medicine, Nanjing, Jiangsu, P. R. China; Department of Colorectal Surgery, The Second Affiliated Hospital of Nanjing University of Chinese Medicine, Nanjing, Jiangsu, P. R. China

## Introduction

**Figure 1:**
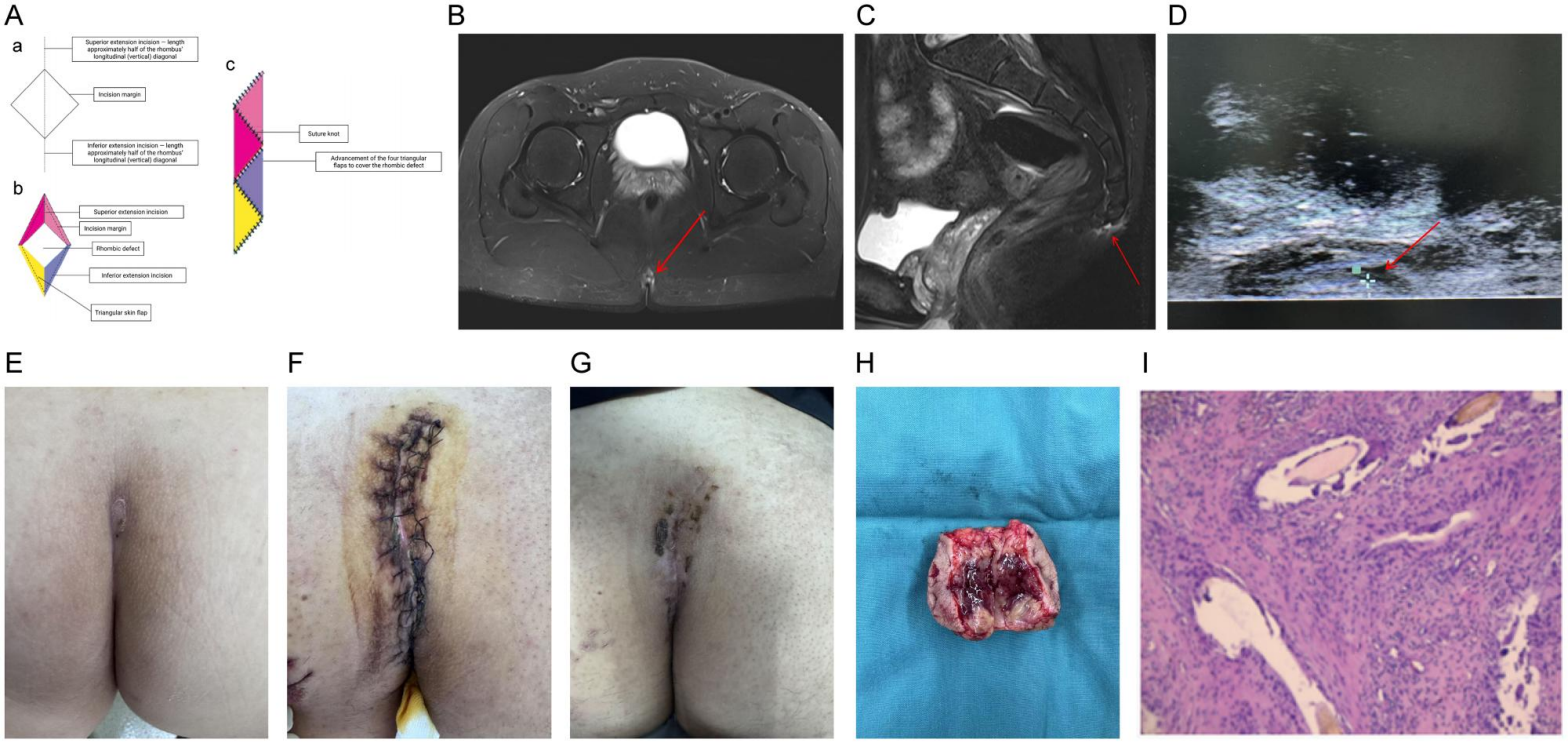
Schematic and clinical images of PSD surgery using the KYF. (A) Diagram of the KYF: (a) planned excision area with superior and inferior extension lines; (b) design of four triangular flaps; (c) final flap position after advancement and closure. (B and C) Pelvic 3.0T MR showing a subcutaneous lesion in the sacrococcygeal region (irregular strip-like T1- and T2-hyperintense signals at the level of the second and third coccygeal segments with mild surrounding soft-tissue edema). (D) Bedside ultrasound demonstrating a hypoechoic subcutaneous area (5.2 × 0.6 cm) with ill-defined borders and internal linear hyperechoic strands, without communication to the anorectal canal. (E) Preoperative appearance of the lesion. (F) Postoperative Day 5 wound appearance. (G) Postoperative Day 14 wound appearance. (H) Gross surgical specimen. (I) Histopathology showing hair shaft-like structures with foreign body giant cell reaction and chronic inflammatory cell infiltration, consistent with PSD (HE staining, ×100).

Complete excision remains the standard treatment for sacrococcygeal pilonidal sinus disease (PSD); however, wide post-excisional defects often render direct midline closure susceptible to dehiscence and recurrence because of high wound tension. Although open healing lowers the risk of recurrence, it entails prolonged wound care and patient discomfort; in contrast, off-midline flaps, such as Limberg, Karydakis, Z-plasty, and V–Y plasty, enhance outcomes by shifting scars and reducing wound tension [1]. However, these flaps can still cause complications, such as blistering, necrosis, seroma, or pain, and often require extensive dissection [2]. To address these limitations, the kiss Y flap (KYF) was developed to minimize undermining and avoid long-distance transposition. Unlike the continuous curved incision in classic S-plasty, the KYF technique achieves low-tension closure by limited advancement of multiple small triangular subunits and offers reliable coverage, thereby promoting smoother healing, reducing complications, and enhancing patient comfort. To our knowledge, this report presents the first clinical application of the KYF technique as a promising and simplified alternative for PSD reconstruction (Fig. 1A).

## Case report

An 18-year-old male patient presented with a 2-year history of intermittent pain and swelling in the sacrococcygeal region, accompanied by occasional skin breakdown and discharge, though without systemic symptoms. Upon examination, a depressed midline sinus opening containing hair was noted, with mild tenderness and minimal discharge on compression. Anoscopic and digital rectal examinations yielded unremarkable results, and no rectal bleeding was observed.

Preoperatively, noncontrast 3.0-T pelvic magnetic resonance imaging (MRI) revealed a subcutaneous lesion in the sacrococcygeal region consistent with a pilonidal sinus (Fig. 1B and C). Bedside ultrasonography demonstrated a 5.2 × 0.6 cm hypoechoic subcutaneous area with poorly defined borders and linear, mildly hyperechoic strands, without connection to the anorectal canal (Fig. 1D). On preoperative probing, the tract measured approximately 2 cm transversely and 5 cm vertically; routine preoperative tests showed no contraindications to surgery. Hair was removed 1 h before surgery using a depilatory cream to minimize the risk of hair embedding and postoperative folliculitis. No mechanical or oral bowel preparation was used. Prophylactic intravenous administration of ceftazidime 2 g twice daily was initiated preoperatively and continued for 48 h postoperatively. Under general anesthesia and with the patient in the prone position, the lesion was probed and outlined for diamond-shaped excision. All diseased tissues were removed down to healthy margins and periosteum. After resection, the diamond-shaped wound was extended by half its long-axis length on the upper and lower sides of the incision, and the full-thickness flap was freed to approximately 1.0 cm beyond the lateral boundaries to allow tension-free forward movement of the flap and closure of the wound edge. After hemostasis and irrigation, a subcutaneous drain was placed, a layered closure was performed, and a vacuum-sealed drainage device was applied for 72 h. Thereafter, daily red-light therapy was applied to the wound to promote microcirculation and healing. Postoperatively, the patient was kept in the prone or lateral position, drains were removed when the output decreased, and sutures were removed at 2 weeks. Normal bowel function was encouraged, and stool softeners were used as needed to avoid straining. Recovery was uneventful, with no necrosis, infection, or hematoma, and complete wound healing was achieved. Preoperative and postoperative appearances are shown in Fig. 1E–G. The surgical specimen and histopathology are shown in Fig. 1H–I.

## Discussion

PSD is a chronic suppurative condition of the sacrococcygeal region, characterized by sinus tract formation, recurrent infections, and purulent discharge, and markedly affects the quality of life [3]. It occurs predominantly in young men, particularly those who are obese, hirsute, or sedentary, and affects 26 per 100,000 people in the USA [4]. The leading hypothesis attributes PSD to friction in the natal cleft, allowing hairs to penetrate the skin, act as foreign bodies, and induce chronic inflammation [5]. Surgical excision remains the only curative treatment; however, traditional methods such as midline closure or open healing are hindered by high wound tension, delayed recovery, and poor cosmetic results. The wound dehiscence rates after midline closure reached 74%, with recurrence up to 45% [6]. Consequently, off-midline flap reconstructions have become the preferred standard for definitive closure in many centers to reduce tension and improve outcomes.

Off-midline techniques such as Limberg, Karydakis, V–Y, and Z-plasty are increasingly preferred for reducing wound tension, hastening healing, and lowering recurrence. In a series of 476 patients, primary midline closure was associated with significantly higher rates of seroma (*P *= 0.006), wound dehiscence (*P *< 0.001), and recurrence (*P *= 0.017) compared with the Karydakis flap, which showed a recurrence advantage [7]. A meta-analysis comparing Limberg and Karydakis flaps revealed a shorter operative time with Karydakis (95%CI: 0.53–13.48, *P *< 0.05) and a difference in seroma rates (95%CI: 0.24–0.56, *P *< 0.05) [8]. Despite improved primary healing, current flap techniques still have some drawbacks: Limberg flaps may cause tension-related complications, whereas Karydakis, Z, and V–Y flaps require extensive mobilization and large subcutaneous pockets, increasing the incidence of trauma, seroma, and pain.

The ideal closure technique for post-excisional PSD wounds should be simple, result in minimal complications, and minimize the risk of recurrence. To our knowledge, this is the first report of the clinical application of the KYF technique, which involves creating four small triangular flaps by extending the superior and inferior ends of the diamond excision. This design allows limited undermining, preserves perfusion, and facilitates low-tension closure with reliable coverage. Advantages include reduced trauma, fewer complications, such as seroma or necrosis, and smaller, cosmetically favorable scars. In our experience, the procedure was rapid and safe and yielded smooth healing without recurrence, indicating KYF as a promising option for PSD reconstruction.

This initial report is limited by a short follow-up period, which is insufficient to assess long-term recurrence, and the absence of direct statistical comparisons with established flaps such as Limberg or Karydakis. Moreover, KYF is best suited for vertically elongated, laterally narrow diamond defects; thus, its applicability varies by shape. Future studies should involve multicenter, adequately powered prospective trials that compare KYF with Limberg, Karydakis, V–Y, and other techniques across various outcomes, such as operative time, complication rates, healing time, recurrence, pain, cosmetic satisfaction, and cost-effectiveness, to define its optimal indications and clinical role.

## Conclusions

In summary, the KYF is a simple, low-tension, and safe novel flap design that shows promise as an additional reconstructive option after complete excision of PSD.

## Authors’ contributions

J.L.L. and H.Y.J. designed the research; J.L.L., X.R.Y., J.W., and Y.Y. were responsible for the definition of intellectual content, literature search, and data acquisition; X.C.Z., J.B.G., C.X.Z., and X.Y.Z. performed the clinical examination and management of the patient; J.L.L. drafted the manuscript; J.L.L., X.C.Z., X.R.Y., and H.Y.J. critically revised the manuscript for important intellectual content. All authors read and approved the final version of the manuscript.
